# Randomized double-blind placebo-controlled trial of hydrogen inhalation for Parkinson’s disease: a pilot study

**DOI:** 10.1007/s10072-021-05489-4

**Published:** 2021-07-28

**Authors:** Asako Yoritaka, Yasuko Kobayashi, Tetsuo Hayashi, Shinji Saiki, Nobutaka Hattori

**Affiliations:** 1 Department of Neurology, Juntendo University Koshigaya Hospital, Fukuroyama 560, Koshigayashi, Saitama 343-0032 Japan; 2 Division of Pharmacology, Juntendo University Koshigaya Hospital, Saitama, Japan; 3 Department of Neurology, Juntendo University School of Medicine, Tokyo, Japan

**Keywords:** Hydrogen gas, Oxidative stress, Parkinson’s disease, Randomized double-blind placebo-controlled trial, Compliance, Adherence

## Abstract

**Background:**

Oxidative stress is involved in the progression of Parkinson’s disease (PD). Recent studies have confirmed that molecular hydrogen (H_2_) functions as a highly effective antioxidant in animal models of PD. A placebo-controlled, randomized, double-blind, parallel-group clinical pilot study was conducted to assess the efficacy of hydrogen gas inhalation in Japanese patients with PD on treatment with levodopa.

**Methods:**

Twenty participants fulfilling the Movement Disorder Society criteria were enrolled. Participants inhaled 6.5 (0.1) vol% hydrogen gas in 2 L/min of mixed air or placebo air for 16 weeks, twice a day for 1 h.

**Results:**

Five participants were excluded due to deviation from the protocol of the total duration of inhalation < 112 h. No significant differences were seen in the change in the total Movement Disorder Society Unified Parkinson’s Disease Rating Scale score from baseline to the 16^th^ week between the group that inhaled hydrogen gas and the group that inhaled placebo air (Mann–Whitney *U* test, *p* > 0.05). No adverse events were seen. The compliance to the protocol-based duration of inhalation time in all participants decreased with the elderly participants, the higher daily dose of levodopa, and the higher PDQ-39 items on emotions (*n* = 20, *p* < 0.05).

**Conclusion:**

This pilot study revealed that the inhalation of molecular hydrogen gas was safe, but did not show any beneficial effects in patients with PD.

**Trial registration:** UMIN ID: 000,039,217 (October 6, 2018)

## Introduction

The increase in iron and lipid peroxidation and the decrease in reduced form of glutathione levels observed in the substantia nigra of patients with Parkinson’s disease (PD) [1] suggest that oxidative stress may play a role in the pathogenesis of PD. Molecular hydrogen (H_2_) has been shown to reduce oxidative stress and dopaminergic neuronal cell loss in a PD model [2]. A randomized, double-blind multicenter trial with drinking H_2_-water for 72 weeks revealed no effects on the Unified Parkinson’s Disease Rating Scale (UPDRS) scores of patients with PD receiving levodopa treatment [3]. In recent times, inhalation of H_2_ gas has been shown to improve the functions of lung on lung donation after cardiac deaths [4, 5]. We conducted a pilot trial of H_2_ gas inhalation among patients with PD on treatment with levodopa.

## Methods

A placebo-controlled, randomized, double-blind, and parallel-group (1:1) clinical trial was performed in our hospital (trial registration, UMIN ID: 000,039,217) according to the Consolidated Standards of Reporting Trials guidelines. The study was approved by the ethics committee of our institution (Koshigaya 30–5). Written informed consent was obtained from all the participants. The inclusion criteria were patients on levodopa treatment who fulfilled the Movement Disorder Society (MDS) criteria of PD [6], were between 1 and 3 of the modified Hoehn and Yahr (H–Y) stage, and between 40 and 80 years of age. Exclusion criteria included cognitive deterioration (score < 25 in mini-mental state examination), presence of lung disease, other serious diseases, and malignant tumors. Anti-parkinsonism drugs were not changed during the trial, including during the 8 weeks before the baseline assessment. Allocation was made sequentially based on the age and H–Y stage by one author (Y.K.) who dispensed the therapy.

The H_2_-producing machine (MHG-2000α) generated 6.5 (0.1) vol % H_2_ gas in 2 L/min of mixed air by electrolysis. Assuming that the participants inhaled 5 L/min of air, 3.0–3.5% hydrogen in 2 L/min of air would be equal to 1.2–1.4% H_2_-air mixture. The placebo air machine was created by disconnecting an electrode for electrolysis, but still produced 2 L/min of air using the air pump. The H_2_ gas and placebo gas machines were indistinguishable. All participants were required to inhale the gas twice a day for 1 h for 16 weeks and had to record the inhalation hours in a journal. The duration of inhalation using the machine was checked when the participants returned after 16 weeks.

Changes in the total MDS-UPDRS scores from baseline to the 8^th^ and 16^th^ week and 24^th^ week after the inhalation period were evaluated. The primary endpoint of the efficacy of this treatment in PD was the change in the total UPDRS score from baseline to the 16^th^ week. The changes in UPDRS part II, UPDRS part III, each UPDRS score, Parkinson’s disease Questionnaire-39 (PDQ-39), and the H–Y stage at these same time-points were noted. At baseline and the 16^th^ week, urinary excretion of 8-hydroxy-2-deoxyguanosine (8-OHdG), which serves as a marker for oxidative stress [7], and N1, N8-diacetylspermidine (DiAcSpd), which could be a diagnostic and surrogate biomarker for PD [8], were also noted.

## Results

Twenty participants were enrolled from February to October 2019, and no adverse events were reported during the study. The self-assessed mean inhalation time mentioned in the patients’ journals was 182.8 ± 48.8 h (standard deviation), and the mean actual inhalation as seen on the machine was 162.5 ± 63.4 h (standard deviation). Five participants (2 in the placebo group, 3 in H_2_ gas group) were excluded due to deviation from the protocol with a total inhalation time < 112 h. The characteristics of the 15 participants with PD (5 women and 10 men) are shown in Table 1; at the baseline visit, the groups were well matched, except for sex and the mean score of PDQ-39.

**Table 1:** Baseline demographics and changes of parameter of Parkinson's disease characteristics and the change from baseline to 16th week

		Placebo group (*n* = 8)	Hydrogen gas group (*n* = 7)	Mann–Whitney *U* test *p*
Modified Hohen and Yahr stage	Mean (SD)	2.5 (0.5)	2.0 (0.6)	> 0.05
Age	Mean (SD)	66.4 (10.3)	65.9 (9.4)	> 0.05
Male: female	*n*	3:5	7:0	
Onset age	Mean (SD)	56.8 (11.8)	56.7 (8.7)	> 0.05
Disease duration (years)	Mean (SD)	10.0 (6.9)	9.1 (7.1)	> 0.05
Levodopa (mg)	Mean (SD)	431.3 (192.7)	371.4 (256.3)	> 0.05
Wearing off +	*n*	3	1	
MDS-UPDRS Total	Mean (SD)	47.8 (23.9)	34.9 (18.6)	> 0.05
Part I	Mean (SD)	7.6 (4.6)	5.4 (2.1)	> 0.05
Part II	Mean (SD)	9.8 (6.5)	5.9 (5.6)	> 0.05
Part III	Mean (SD)	28.8 (14.8)	22.0 (10.6)	> 0.05
Part IV	Mean (SD)	1.6 (2.1)	1.6 (2.8)	> 0.05
PDQ-39	Mean (SD)	48.9 (25.9)	22.4 (19.9)	0.021
Urine N1,N8-diacetylspermidine (ng/mgCr)	Mean (SD)	9.4 (3.0)	9.4 (2.4)	> 0.05
N1,N8-diacetylspermidine (pmol/100 µl serum)	Mean (SD)	0.38(0.16)	0.31(0.06)	> 0.05
Inhalation time Hours	mean (SD)	203.1 (23.0)	185.0 (26.1)	> 0.05
Change from the baseline to the 16^th^ Week				
MDS-UPDRS Total	Mean (SD)	0.8 (10.3)	1.9 (13.1)	> 0.05
Part I	Mean (SD)	0.0 (3.8)	-0.1 (2.8)	> 0.05
Part II	Mean (SD)	0.9 (2.1)	2.4 (4.0)	> 0.05
Part III	Mean (SD)	–1.8 (4.7)	3.0 (9.0)	> 0.05
PDQ-39	Mean (SD)	–6.9 (24.7)	4.9 (8.7)	> 0.05
Urine 8-hydroxy-2-deoxyguanosine (ng/mgCr)	Mean (SD)	–0.3 (4.8)	0.4 (2.1)	> 0.05
N1,N8-diacetylspermidine (pmol/100 µl serum)	Mean (SD)	-0.01(0.08)	0.04(0.08)	> 0.05

*MDS-UPDRS* Movement Disorder Society Unified Parkinson’s disease rating scale, *PDQ*-*39* Parkinson’s disease Questionnaire-39

No significant differences were seen in the change in the total UPDRS score from baseline to the 16th week between the H_2_ gas and placebo air groups (Mann–Whitney *U* test, *p* > 0.05; Table 1). No significant differences were seen in the changes of the scores on parts II and III or the individual parts of the MDS-UPDRS, PDQ-39 score, DiAcSpd, or urine 8-OHdG between the two groups.

## Discussion

This pilot study revealed that the inhalation of H_2_ gas was safe but had no beneficial effects in patients with PD. In our previous study, the inhalation of H_2_ gas twice a day for 10 min for 4 weeks significantly increased the urinary excretion of 8-OHdG, a marker for oxidative stress, by 16% [9]. However, in this study, despite an increase in the duration of inhalation, no significant increase of 8-OHdG was observed compared with the previous study.

The medical adherence rate to the study protocol in PD depended on the dosing frequency [10]. The protocol compliance evaluated by the inhalation time mentioned in the participants’ journals, and the actual inhalation time as noted from the inhalator decreased with the elderly participants, the higher daily dose of levodopa, and the higher PDQ-39 items on emotions (*n* = 20, *p* < 0.05).

There are some limitations to this study. The first is the small number of patients included. Moreover, since five participants were excluded, the final sample size decreased further, and the study was underpowered to conclude the significance (*β* error) of the outcomes. Larger studies could possibly lead to different results. Second, progressed PD stages were not included, and the UPDRS scores in the “off” phase were not evaluated. Third, the compliance to the protocol was low; however, this impact of this factor on the analysis was eliminated by using the count of inhalation time.

In conclusion, the inhalation of H_2_ gas is safe, but it did not show beneficial effects in patients with PD. Further development and application methods are required to accelerate effective treatments for PD.

## Data Availability

The datasets generated during and/or analyzed during the current study are available from Asako Yoritaka on reasonable request.
